# Effects of Fermented *Houttuynia cordata* Thunb. on Diabetic Rats Induced by a High-Fat Diet with Streptozotocin and on Insulin Resistance in 3T3-L1 Adipocytes

**DOI:** 10.1155/2021/6936025

**Published:** 2021-08-06

**Authors:** Wannachai Sakuludomkan, Ranchana Yeewa, Subhawat Subhawa, Chakkrit Khanaree, Arisa Imsumran Bonness, Teera Chewonarin

**Affiliations:** ^1^Department of Biochemistry, Faculty of Medicine, Chiang Mai University, 110 Intravaroros Road, Sripoom, Muang, Chiang Mai 50200, Thailand; ^2^The School of Traditional and Alternative Medicine, Chiang Rai Rajabhat University, 80 Phaholyothin Road, Ban Du, Muang, Chiang Rai 57100, Thailand

## Abstract

*Houttuynia cordata* Thunb. (*plukaow* in Thai language) exhibits several biological properties, and many products of *H. cordata* are therefore commercially available for human consumption, such as fermented juice or tablets as food supplements. This study aimed to investigate the antidiabetic effects of fermented *H. cordata* (HC) in high-fat diets and streptozotocin-induced diabetic rats. Oral administration of HC at a dose of 100 mg/kg.bw not only maintained bodyweight, food intake, and water consumption but also reduced blood glucose levels and improved glucose tolerance ability in the diabetic rats. Moreover, HC also decreased oxidative stress markers in serum and inflammatory-related mediators in pancreas tissues, indicating the improvement of pancreatic beta-cell function in the diabetic rats. In order to clarify the mechanism of HC, the effects of ethanolic extract of HC (HCE) on insulin resistance were determined in 3T3-L1 adipocytes. FHE could recover glucose uptake and decrease lipolysis in palmitate-treated 3T3-L1 adipocytes. Taken together, these results demonstrate that HC can improve diabetic symptoms by enhancing insulin sensitivity, reducing oxidative stress, and suppressing inflammation.

## 1. Introduction

Type 2 diabetes mellitus is dramatically increasing worldwide and causes morbidity and mortality, as well as economic burdens on countries [1]. Epidemiologic studies have demonstrated the relationship between an increased incidence of type 2 diabetes and obesity-associated insulin resistance [2]. High accumulation of fat in adipose tissue plays an important role in chronic low-grade inflammation, leading to an increase in proinflammatory cytokine production [3]. These proinflammatory cytokines can also reduce insulin sensitivity in adipocytes, which cause the release of free fatty acids into the bloodstream [4–6]. Consequently, free fatty acids, especially palmitate, can induce muscle cells and liver cells to become insulin resistant, resulting in hyperglycemia [7, 8]. The goal of treating diabetes as a medical condition is to reduce blood glucose levels, which can prevent or delay the occurrence of complications relating to the disease [9]. Nevertheless, common types of oral medication used for the treatment of diabetes have demonstrated side effects and caused adverse reactions [10]. Currently, many herbal medicines have been recommended for the prevention and treatment of diabetes, in addition to conventional medication [11].

*Houttuynia cordata* Thunb., commonly known in Thai as plukaow, is a natural herb indigenous to local areas of Northern Thailand that is used in cooking in the region. It has been recognized in folk medicine for being used to control bacterial [12], viral infections [13], diuresis [14], antiallergic [15], antiobesity [16], anticancer [17], and antidiabetic activities [18]. Moreover, it contains many constituents, including polyphenols (chlorogenic acid, rosmarinic acid, vanillic, and protocatechuic acid), flavonoids (quercetin, quercitrin, rutin, and hyperin), alkaloids, essential oils, organic acids, and microelements [19]. Nowadays, in Thailand, alternative herbal products are widely used as dietary supplements. The fermentation of *H. cordata* using natural microorganism has produced an interesting natural product, with tablet forms subsequently developed for commercial production. It has been reported that fermentation of *H. cordata* is able to increase its phytochemical composition, such as flavonoid compounds, beta-glucan, and organic acid, and also improve the biological activities through biotransformation [20, 21].

Therefore, this study aimed to investigate the effects of fermented *H. cordata* tablets on diabetic rats induced by a high-fat diet with streptozotocin. Moreover, the underlying mechanisms of fermented *H. cordata* extract on insulin resistance were then investigated in palmitate-treated 3T3-L1 adipocytes.

## 2. Materials and Methods

### 2.1. Material Preparation

Fermented *H. cordata* tablets (HC) in this study were obtained from Rincome Group Company limited, Doi Saket District, Chiang Mai Province, Thailand. HC was finely ground by mortar and pestle, and then the powder was suspended with 0.5% carboxymethylcellulose (CMC) in distilled water at a concentration of 10 mg/ml and 100 mg/ml. The suspension of HC was then used in rat experimentation. For *in vitro* study, 10 g of HC powder was extracted by using either 80% ethanol (100 ml) or distilled water overnight at room temperature. After being filtered and evaporated, the concentrated crude ethanol and water extract were lyophilized to obtain the powdered HC ethanol extract (HCE) and water extract (HCW), respectively.

### 2.2. Determination of Phenolic Contents and Flavonoid Contents

The total phenolic contents of the HCE and HCW were estimated by using Folin–Ciocalteu reagent assay [22], calculated by gallic acid standard curve and expressed in terms of milligrams of gallic acid equivalents per gram of extract. The total flavonoid contents were determined by using aluminium chloride colorimetric assay [23], calculated by catechin standard curve and expressed in terms of milligrams of catechin equivalents per gram of extract.

### 2.3. Identification and Quantitation of Major Constituents in HCE and HCW by HPLC Fingerprint Analysis

The phenolic and flavonoid compounds in both extracts were determined by HPLC according to the method of Pintha et al. [24]. Briefly, the solution was filtered through 0.2 *μ*m and then subjected to separation by the ODS-3-C18 column (4.6 mm × 250 mm, 5 *μ*m particle diameter). The 0.1% trifluoroacetic acid (TFA) in methanol and methanol was used as mobile phases A and B, respectively. The gradient was performed as follows: 0–35 min, 90–10% mobile phase A and 10–90% mobile phase B; 35–40 min, 10–90% mobile phase A and 90–10% mobile phase B with a flow rate of 1.0 ml/min and injection volume of 10 *μ*l. The phenolic and flavonoid absorption peaks were measured by a UV detector with wavelengths at 280 and 320 nm, respectively. Peak area and retention time of the compounds in the extract were compared to phenolic and flavonoid standards including chlorogenic acid, gallic acid, catechin, and quercetin (Sigma-Aldrich, USA); caffeic acid, rosmarinic acid, luteolin, rutin, and apigenin (Chengdu biopurify phytochemicals, China); vanillic acid, and ferulic acid (ChromaDex, USA).

### 2.4. Total Beta-Glucan Analysis

Total beta-glucan content was determined by using Congo red colorimetric method [25]. Two microliters of the extract dissolved in DMSO was added to Congo red reagent (0.08 mg/mL in phosphate buffer pH = 7). The reaction mixture was incubated in dark conditions at room temperature for 30 min, and then the absorbance was measured at 510 nm using a spectrophotometer. The beta-glucan contents in the extracts were calculated by being compared to the *β*-D-glucan standard and expressed as mg/g extract.

### 2.5. Determination of Anti-DPPH and ABTS Free Radical Capacity

The free radical scavenging activity of HCE was determined by using DPPH stable radicals according to the method of Brand-Williams et al. [26]. The ABTS^•+^ radical was performed according to the method of Kumar et al. [27]. The results of scavenging inhibition of DPPH and ABTS^•+^ were presented as the concentration of the extracts, which scavenged free radicals by 50% (SC_50_).

### 2.6. Study of HC in Diabetic Rats Induced by a High-Fat Diet with Streptozotocin

Eight-week-old male Wistar rats (250–300 g) were purchased from the National Laboratory Animal Center, Mahidol University, Thailand (NLAC-MU). Rats were housed at 25°C (air-conditioned) with 12 : 12 hrs-light/dark cycles (lights were turned on from 06.30–18.30). Rats were fed a normal laboratory pellet diet and water *ad libitum*.

**Figure 1:**
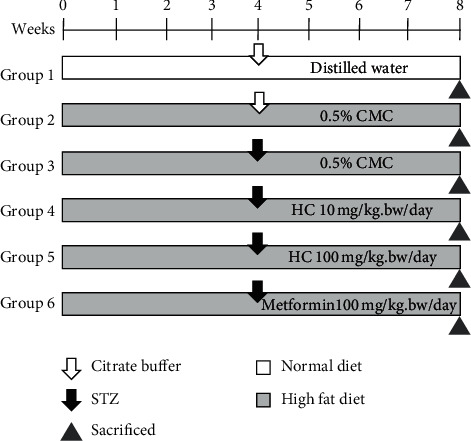
Experimental protocols of HFD-STZ-induced diabetic rat model. Each group contain 6 rats. CMC, carboxymethylcellulose; HC, the suspension of fermented *H. cordata*. tablet.

As shown in Figure 1, 6 rats were fed a standard laboratory chow throughout the duration of the experiment and assigned to the normal control group (Group 1). To establish diabetic rats, the other 30 rats were fed a high-fat diet (HFD) containing (50% fat, 25% carbohydrate, and 25% protein). After 4 weeks, 24 rats of HFD were intraperitoneally injected with one dose of streptozotocin at 35 mg/kg.bw/ml dissolved in 0.1 m citrate buffer pH 4.5, while the remaining 6 rats of HFD group and the normal control rats (group 1) were injected with citrate buffer pH 4.5. Seventy two hours after the streptozotocin injection, the fasting blood sample of each rat was collected from tail vein, and then glucose levels were measured by a glucose meter (Easy G, OPTIMA, Taiwan). The hyperglycemic rats, which showed blood glucose levels in excess of 250 mg/dl, were grouped together for further experiment. Rats in groups 2–6 were continued to be fed a high-fat diet and water *ad libitum*. Rats in HFD control (group 2), which were injected with citrate buffer, were orally administrated 0.5% CMC. Hyperglycemic rats in group 3 (HFD-STZ control) were orally fed 0.5% CMC, while rats in groups 4–6 were fed HC 10 mg/kg.bw, HC 100 mg/kg.bw and 100 mg/kg.bw metformin, respectively. The oral administration in each condition was performed in the morning once a day for 4 weeks. Rat bodyweight, fasting blood glucose levels, food intake, and water consumption were recorded weekly.

### 2.7. Oral Glucose Tolerance Test in Rats

At the end of the experiment, all rats were fasted for more than 10 hours. Blood samples were collected to determine blood glucose levels at the baseline level (time-0). Then, the rats were orally administrated 2 g/kg.bw of glucose, and blood samples were taken from the tail vein of each rat at 30, 60, 90, and 120 min in order to measure the blood glucose using a glucose meter. The average blood glucose level of each group (*y*-axis) was plotted against time of blood collection (*x*-axis). The OGTT curve of each group was established, and the area under the curve (AUC) was calculated by GraphPad Prism 6.0 (Graph Pad Software, San Diego, CA, USA).

### 2.8. Blood and Organ Sampling

All rats were fasted overnight and then sacrificed at week 9 of the experiment. Blood samples were collected from hepatic veins and centrifuged. Serum was separated and kept at −80°C for biochemical analysis. The pancreas was removed for histopathological examination and determination of the proinflammatory related cytokines and enzyme expression.

### 2.9. Biochemical Analysis

Total cholesterol, total triglyceride, and HbA1c levels were determined using automatic machines at the BT LAB Medical Laboratory, Chiang Mai, Thailand. Malondialdehyde (MDA) levels were estimated by TBARS assay, according to the method of Chaiyasut et al. [28]. Inflammation was determined by measuring serum nitrite, the ultimate product of nitric oxide, according to the method of El-Shakour et al. [29].

### 2.10. Fasting Serum Insulin Levels (FINS) and Homeostasis Model Assessment of Insulin Resistance (HOMA-IR) Analysis

HOMA-IR is a method used to determine insulin resistance and beta-cell function, which are calculated from the baseline fasting blood glucose and fasting insulin levels or C-peptide concentrations by using an ELISA kit according to the manufacturer's instruction (Merck Millipore, Germany) [30]. Fasting insulin levels were determined by establishing insulin standard curve and expressed as (ng/ml). HOMA-IR was calculated using the following formula:

(1) $$\begin{array}{c} \text{HOMA}-\text{IR}=\frac{\text{fasting insulin level}\left(\text{ng}/\text{ml}\right)\times \text{fasting glucose level}\left(\text{mg}/\text{dl}\right)}{405.1}. \end{array}$$

### 2.11. Histological Determination

Formalin-fixed paraffin-embedded pancreas tissue was sectioned and stained with hematoxylin and eosin (H & E). Pancreas histology and Islet of Langerhans architecture were defined and photographed under an optical microscope (40x). The area of Islet of Langerhans was calculated by AxioVision LE64 program and expressed as (%) of islet area. The number of Islets of Langerhans/pancreas section was counted using at least three fields of view and represented as mean ± SD.

### 2.12. Detection of Proinflammatory-Related Cytokine and Enzyme Expression in Pancreas Tissue by RT-PCR

Frozen pancreatic tissue was homogenized in 1 ml of TRIzol chloroform. Total RNA was reverse-transcribed to cDNA by using high capacity RNA-to-cDNA kit (ReverTra Ace qPCR RT) master mix (TOYOBO, Japan), according to the manufacturer's instruction. To determine the copy number of specific target cDNA, the 20 *μ*L of mixture solution contained 1 *μ*g cDNA, 2x sensiFAST™ SYBR® Lo-ROX, forward and reverse primers, and Nuclease-free water was subjected to ABI 7500 real-time PCR system. Primer sequences for rats are listed as follows: TNF-*α*, 5′-AAATGGGCTCCCTCTCATCAGTCC-3′ (forward) and 5′-TCTGCTTGG TGGTTTGCTACGAC-3′ (reverse); IL-6, 5′-TCCTACCCCAACTTCAATGC TC-3′ (forward) and 5′-TTGGATGGTCTTGGTCCTTA GCC-3′ (reverse); *IL-*1*β*, 5′-CAC CTCTCAAGCA GAGCACAG-3′ (forward) and 5′-GGGTTCCATGGTGAAGTCAAC-3′ (reverse); *iNOS,* 5′-CATTGGAAGTGAAGCGTT TCG-3′ (forward) and 5′-CAGCTGGGC TGTACAAACCTT-3′ (reverse); *COX-*2, 5′-GCCCACCAACTTACAATGTG C-3′ (forward) and 5′-CATGGGAGTTG GGCAGTCAT-3′ (reverse); *GAPDH,* 5′-GACATGCCG CCTGGAGAAAC-3′ (forward) and 5′-AGCCCAGGATGCCCTTTAGT-3′ (reverse). The setup temperatures and times were as follows: 95°C for 10 min, followed by 40 cycles of 95°C for 15 sec and 60°C for 1 min. Results were normalized to GAPDH and expressed relative to the control cells.

### 2.13. Cell Culture and Cell Differentiation

3T3-L1 preadipocytes from ATCC were cultured in DMEM containing 10% fetal bovine serum (FBS) and 1% penicillin/streptomycin solution in a humidified atmosphere at 37°C in a CO_2_ incubator at 5% CO_2_. At two days after-confluence (designated as day 0), cell differentiation was induced by culturing in DMEM containing 0.5 mM isobutylmethylxanthine (IBMX), 0.1 *μ*M dexamethasone, 1.67 *μ*M insulin, and 10% fetal bovine serum for 3 days. The differentiated cells were then incubated in DMEM containing 10% FBS and 1.67 *μ*M insulin for 10 days. The differentiated states of 3T3-L1 adipocytes were determined by fat droplet accumulation using oil red O staining and observation under a microscope. Next, the matured adipocytes were cultured for further experiments.

### 2.14. 2-NBDG Uptake and Lipolysis in Palmitate-Treated Cells

To determine the effects of HCE on palmitate-induced insulin resistance, mature adipocyte was cultured in DMEM containing 750 *μ*M palmitate (in BSA solution) together with various concentrations of HCE or 1 mM of metformin (positive control) for 16 hours without insulin. After that, the conditioned cells were washed with phosphate buffer saline (PBS) and continuously treated with HCE or metformin for 24 hours without insulin.

Palmitate-treated cells were then washed and cultured in low glucose DMEM for 3 hours at 37°C. After that, the conditioned cell medium was replaced by a medium containing 100 mM 2-NBDG (glucose analog) and 100 mM of insulin for 1 hour at 37°C in a CO_2_ incubator. After washing the excess of 2-NBDG, intracellular fluorescence intensity of 2-NBDG-glucose was measured at 485 nm and 535 nm using a microplate fluorescence spectrophotometer. The alteration of glucose uptake was calculated by comparing fluorescence intensity levels to the control and then expressed as a percentage. Lipolysis in adipocytes was determined by free glycerol released into the culture medium after treatment, which was measured by an adipolysis assay kit (Cayman Chemical, USA). The glycerol content was calculated by glycerol standard curve and expressed as a percentage from nontreated cells.

### 2.15. Statistical Analysis

Data is presented as mean ± standard deviation (SD). Statistical significance in all data was determined by one-way ANOVA followed by Tukey's multiple comparisons, while Student's *t*-test was used to compare the difference between the two experimental groups using GraphPad Prism software version 6. *p* < 0.05 was considered statistically significant.

## 3. Results

### 3.1. Effect of High-Fat Diet and Streptozotocin on Rat Characteristics

**Figure 2:**
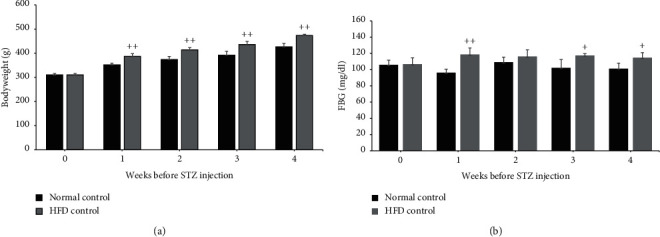
Effect of HFD on rat characteristics. (a) Bodyweight and (b) fasting blood glucose in normal and high-fat diet groups after 4 weeks of consumption. ^+^*p* < 0.01 and ^++^*p* < 0.001 versus normal control group. Results are presented as means ± SD (*n* = 6 each group).

**Figure 3:**
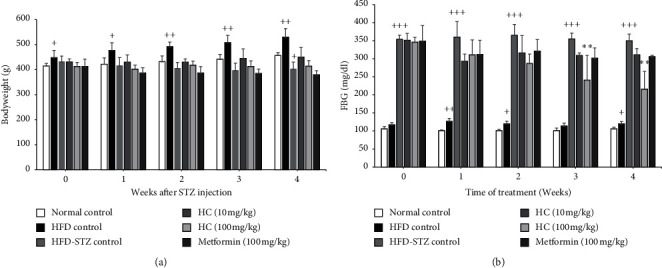
Effect of HC on rats' bodyweight and fasting blood glucose. (a) Weekly bodyweight and (b) fasting blood glucose (FBG) level of each group. ^+^*p* < 0.05, ^++^*p* < 0.01, and ^+++^*p* < 0.001 versus normal control group. ^*∗∗*^*p* < 0.0001 versus HFD-STZ control group. Results are presented as means ± SD (*n* = 6 each group).

The consumption of a high-fat diet (HFD) for 4 weeks resulted in a significant increase in the rats' bodyweight and a decreased relative pancreas weight compared to the normal diet-fed rats (Figure 2(a) and Table 1). In addition, fasting blood glucose (FBG) levels of the HFD-fed rats were significantly higher than those of the normal diet-fed rats (Figure 2(b)), consistent with increasing food intake and water consumption (data not shown). Notably, streptozotocin (STZ) treatment in HFD-fed rats decreased the bodyweight (Figure 3(a)). Besides, the STZ injection also led to a significantly increased FBG level, approximately 3.5- and 3-fold compared to the normal control group and only-HFD-fed group, respectively (Figure 3(b)). Moreover, the relative weight of the rats' liver significantly increased (*p* < 0.001) in the HFD-STZ control group compared to the normal control group (Table 1). These results suggest that HFD consumption contributes to mild insulin resistance, while the combination of HFD with STZ injection could encourage the onset of symptoms of diabetes in these experimental rats.

### 3.2. Effect of HC on Bodyweight, Organ Weight, FBG, Food Intake, and Water Consumption in HFD-STZ-Induced Diabetic Rats

Administration of fermented *H. cordata* tablet (HC) at a dose of 10 mg/kg.bw and 100 mg/kg.bw could maintain the bodyweight of the rats when compared to the HFD-STZ control group (Figure 3(a)). In addition, administration of metformin at a dose of 100 mg/kg.bw dramatically decreased the bodyweight of rats compared to the HFD-STZ control group (*p* value = 0.49) (Figure 3(a)). At weeks 3 and 4 of the experiment, HC at a dose of 100 mg/kg.bw significantly suppressed FBG in the diabetic rats, which corresponds with the characteristics of the diabetic rats, including the increased food intake and water consumption (Figure 3(b) and Table 2). Moreover, administration of HC at a dose of 100 mg/kg.bw significantly increased the pancreas weight compared to the HFD-STZ control group (Table 1). These results indicate that HC attenuated the symptoms of diabetes in the HFD-STZ-induced rat model.

### 3.3. Effect of HC on Glucose Tolerance in HFD-STZ-Induced Diabetic Rats

**Figure 4:**
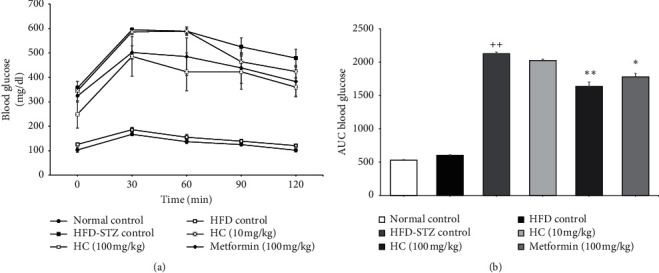
Effect of HC on glucose tolerance in diabetic rats. (a) Oral glucose tolerance test curves of rats determined during the last weeks of the experiment; (b) area under the curve. ^++^*p* < 0.001 versus normal control group. ^*∗*^*p* < 0.001 and ^*∗∗*^*p* < 0.001 versus HFD-STZ control group. Results are presented as means ± SD (*n* = 6 each group).

HFD-STZ control group showed higher blood glucose levels at 0, 30, 60, 90, and 120 min after drinking glucose compared to the normal control group. However, blood glucose levels in 100 mg/kg.bw of HC fed rats were lower than those of HFD-STZ rats (Figure 4(a)). To compare the modulating effect on glucose tolerance, the area under the curve was calculated and diagrammed (Figure 4(b)). HFD-STZ group showed a significantly higher AUC when compared to the normal control group, while high doses of HC were shown to decrease the AUC by approximately 23%, which were more potent than metformin administration. These results indicate that HC could improve glucose tolerance in HFD-STZ-induced diabetic rats.

### 3.4. Effect of HC on Glucose Utilization Parameters in HFD-STZ-Induced Diabetic Rats

The levels of FBG, fasting Serum Insulin Levels (FINS), homeostasis model assessment of insulin resistance (HOMA-IR), and HbA1c in each group are summarized in Table 3. Interestingly, rats only fed with a high-fat diet exhibited increased serum insulin and blood glucose levels when compared to the normal control group. The results demonstrate that the consumption of high-fat diet could mildly induce insulin resistance in the rats. As a positive control, the HOMA-IR value in HFD-STZ group was significantly increased when compared to the normal control group, which was generated from the decreasing serum insulin level increase blood glucose levels. Unfortunately, the HOMA-IR value was not shown to change in rats that were administrated HC both 10 and 100 mg/kg.bw when compared to the HFD-STZ control group. It can be interpreted that the administration of HC significantly decreased FBG in rats by increasing insulin levels but not by improving insulin sensitivity. In addition, HbA1c was measured to confirm hyperglycemia in the previous period before sacrifice. Concomitant to a high serum glucose level, HFD-STZ rats presented high levels of HbA1c when compared to the normal control group. Conversely, administration of HC at a concentration of 100 mg/kg.bw tended to decrease HbA1c compared to HFD-STZ-treated rats. These results demonstrate the antidiabetic activity of HC in the rat model.

### 3.5. Effect of HC on Dyslipidemia in HFD-STZ-Induced Diabetic Rats

Serum levels of total triglycerides (TG) were clearly higher in HFD-STZ-treated rats compared to the normal control group, while total cholesterol (TC) was slightly different but failed to reach a statistically significant difference (Table 4). Only the elevation of TG was related to hyperglycemia in diabetic rats (Figure 3(b)); therefore, the level of TC was not observed in each treatment compared to normal and HFD-HTZ groups. However, administration of only HC at a dose of 100 mg/kg.bw could decrease TG to a greater degree than metformin when compared to the HFD-STZ control group.

### 3.6. Effect of HC on Pancreas Histological Appearance in HFD-STZ-Induced Diabetic Rats

**Figure 5:**
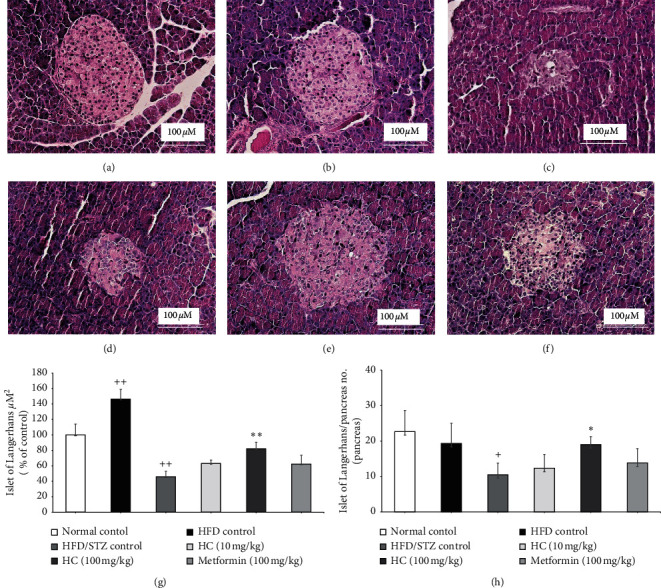
Histological analysis of pancreatic tissue sections stained with H&E. (a) Normal control; (b) HFD control; (c) HFD-STZ control; (d) HC 10 mg/kg; (e) HC 100 mg/kg; (f) metformin; (g) Islet of Langerhans area; (h) Islet of Langerhans/pancreas number. Magnitude 40x. ^+^*p* < 0.01 and ^++^*p* < 0.001 versus normal control group. ^*∗*^*p* < 0.05 and ^*∗∗*^*p* < 0.001 versus HFD-STZ control group. Results are presented as means ± SD (*n* = 6 each group).

Histological change in pancreas tissue was evaluated by H&E staining; the representative figures are shown in Figure 5. The area of Islet of Langerhans in the HFD group was significantly increased when compared to the normal control group, while inflammatory cell infiltration and necrotic cell death were observed in the pancreas of the HFD-STZ-treated group. Additionally, the Islet of Langerhans area and number of Islets of Langerhans/pancreas section in HFD-STZ-treated rats decreased, as shown in Figures 5(g) and 5(h). In contrast, the HFD-STZ rats with HC administration significantly increased in terms of Islet of Langerhans area and number of Islets of Langerhans/pancreas section compared to the HFD-STZ control group. These findings indicate that HC could ameliorate the pathological changes in rat pancreas.

### 3.7. Effects of HC on Inflammation and Oxidative Stress in HFD-STZ-Induced Diabetic Rats

**Figure 6:**
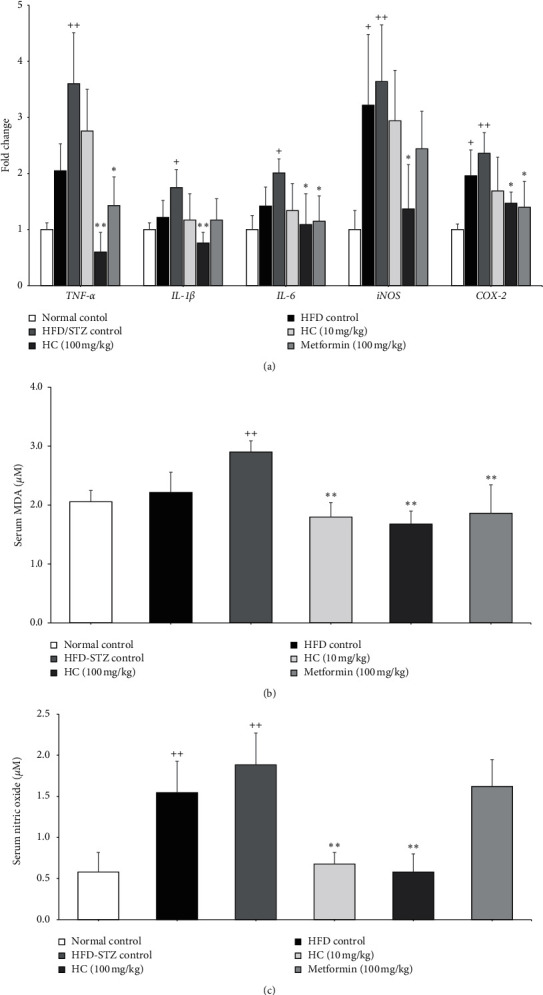
Anti-inflammatory and antioxidant properties of HC in HFD-STZ-induced diabetic rats. (a) Fold change of *TNF-α, IL-*1*β, IL-*6*, iNOS,* and *COX-*2 mRNA level in pancreas tissues of HFD-STZ-treated rats; (b) Serum malondialdehyde (MDA) level; (c) serum nitric oxide. ^+^*p* < 0.01 and ^++^*p* < 0.001 versus normal control group. ^*∗*^*p* < 0.01 and ^*∗∗*^*p* < 0.001 versus HFD-STZ control group. Results are presented as means ± SD (*n* = 6 each group).

To investigate the anti-inflammatory effect of HC in pancreas tissue, mRNA level of inflammatory-related cytokines and key producing enzymes was measured by quantitative real-time PCR (Figure 6(a)). The expression of *TNF-α*, *IL-*1*β*, *IL-*6, *iNOS*, and *COX-*2 mRNA was significantly increased in HFD-STZ-treated rats compared to the normal control, whereas HFD-fed rats were seen to have increased only *iNOS* and *COX-*2 mRNA levels. Interestingly, administration of HC at a dose of 100 mg/kg.bw could significantly decrease mRNA levels of *TNF-α*, *IL-*1*β*, *IL-*6, *iNOS*, and *COX-*2 compared to the HFD-STZ control group. Importantly, the decline of these mRNA levels in the group fed a HC dose of 100 mg/kg.bw was more obvious than that as seen in the metformin group. From these results, it can be suggested that HC treatment could decrease pancreas inflammation of HFD-STZ-treated rats by decreasing the expression of proinflammatory cytokines and enzymes.

As shown in Figure 6(b), serum MDA was significantly increased in the HFD-STZ control group, which represented the increased lipid peroxidation when compared to the normal control group. Consistent with the expression of iNOS (Figure 6(a)), serum nitric oxide was significantly increased in HFD alone and HFD-STZ fed rats when compared with the normal control (Figure 6(c)). Interestingly, administration of HC at a dose of 10 mg/kg.bw and 100 mg/kg.bw significantly decreased MDA levels by approximately 30% and 40%, while nitric oxide levels decreased by approximately 63% and 68% from the HFD-STZ control group, respectively (Figures 6(b) and 6(c)). Moreover, the effect of HC at a dose of 100 mg/kg.bw on suppressing MDA and nitric oxide levels was more pronounced than that of metformin. These data suggest that HC treatment alleviated oxidative stress induced by inflammation that could eventually lead to the recovery of pathologic lesions of the pancreas and improve diabetic symptoms in the rats.

### 3.8. Phytochemical Compositions and Antioxidant Capacity of HC Extracts

**Figure 7:**
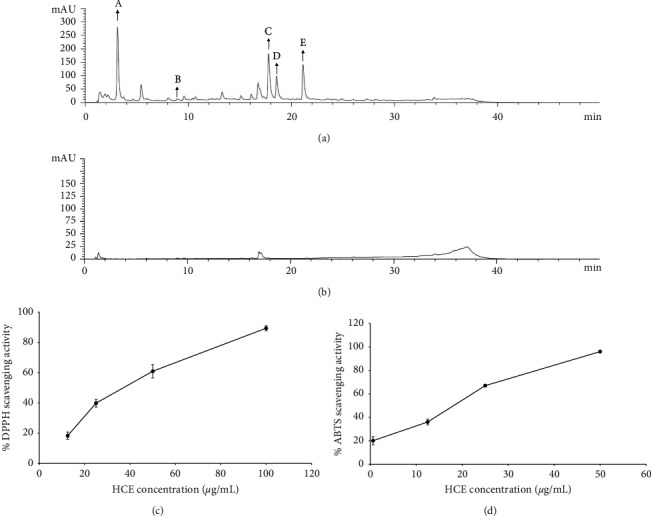
HPLC chromatogram of HC extract. (a) HCE. “A” stands for gallic acid; “B” stands for chlorogenic acid; “C” stands for rutin; “D” stands for rosmarinic acid; “E” stands for quercetin. (b) HCW. The percentage of antioxidant of HCE against (c) DPPH and (d) ABTS free radicals. The results represent mean ± SD of triplicate independent experiments, and each point was significantly different (*p* < 0.05).

To clarify the mechanism of HC, ethanol extract (HCE) and water extract (HCW) of HC were used for analysis. The total phenolic acid and flavonoid contents of HCE were 99.54 ± 2.81 mg gallic acid equivalent/g extract and 50.19 ± 2.83 mg catechin equivalent/g extract, respectively. The total phenolic acid content in HCW was 18.5 ± 0.7 mg gallic acid equivalent/g extract, whereas flavonoids were not found in HCW. HPLC fingerprints of HCE and HCW are shown in Figures 7(a) and 7(b). Phytochemical compounds, including gallic acid, chlorogenic acid, rosmarinic acid, rutin, and quercetin, were found in HCE (12.22 ± 0.02, 1.15 ± 0.01 4.51 ± 0.01, 8.82 ± 0.01, and 7.54 ± 0.01 mg/g extract, respectively). Moreover, the beta-glucan content in HCE was 417.5 ± 0.15 mg/g extract, which is 4.7 times higher than that in HCW (88.76 ± 8.76 mg/g). The concentrations of HCE that was able to scavenge DPPH and ABTS free radicals at 50% (SC_50_) were 37.88 ± 0.66 *μ*g/ml and 18.12 ± 0.37 *μ*g/ml, respectively (Figures 7(c) and 7(d)). Therefore, HCE, which contains a higher amount of phenolic, flavonoids, and beta-glucan, was selected for further investigating the mechanism of HC in the *in vitro* assay. Focusing on the ethanol extract of powdered HC tablet, ten grams of HC could be extracted to 580 mg of HCE. It could be calculated that 10 and 100 mg/ml of HC contained 0.58 and 5.8 mg of HCE, respectively. Therefore, the concentration of HCE in rat feeding doses was 0.58 and 5.8 mg/ml, respectively.

### 3.9. Effect of HCE on Palmitate-Induced Insulin Resistance in 3T3-L1 Adipocytes

**Figure 8:**
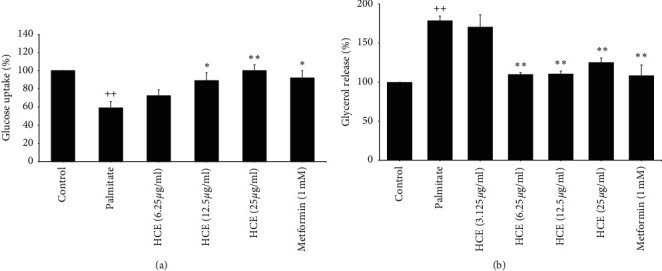
Effect of HCE on insulin resistance in 3T3-L1 adipocytes. (a) Glucose uptake and (b) lipolysis in palmitate-treated 3T3-L1 adipocytes. ^++^*p* < 0.001 versus normal control group. ^*∗*^*p* < 0.01 and ^*∗∗*^*p* < 0.001 versus palmitate-treated group.

Nontoxic concentrations of FHE were used to study the effect of HCE on insulin resistance in 3T3-L1 adipocytes. 750 *μ*M of palmitate could impair insulin sensitivity in 3T3-L1 adipocytes by decreasing 2NBDG uptake, down to 41%, and increasing lipolysis, up to 179%, compared to non-treated cells. Interestingly, HCE at doses of 6.25, 12.5, and 25 *μ*g/ml could restore 2-NBDG uptake to 72, 89, and 100% (Figure 8(a)) and decreased lipolysis by approximately 110, 111, and 125% (Figure 8(b)), respectively. Otherwise, 1 mM metformin could restore 2-NBDG uptake to 92% and decrease lipolysis down to 108%. These findings demonstrate that FHE reduces insulin resistance in palmitate-treated-3T3-L1 adipocytes.

## 4. Discussion

Traditional herbal medicines are nowadays used to prevent, pretreat, or cure diabetes, in addition to common over-the-counter and prescription drugs. Besides, dietary supplements and functional beverages are promising research topics in the field of health products. *H. cordata* is widely used in Thailand in regional cuisine, and its fermented product is produced commercially as tablets for diabetic prevention. In this research, fermented *H. cordata* tablet (HC) was therefore selected to be investigated for its antidiabetic activities.

Obesity is a crucial factor involved in insulin-resistant development. Previous studies have found that rats fed a high-fat diet developed insulin resistance depending on the accumulated fat percentage [31]. Srinivasan et al. also revealed that rats fed a high-fat diet for only 2 weeks resulted in a significant increase in bodyweight, as well as blood glucose, lipoproteins, and insulin levels [32]. Consistently, in this study, rats that were given a high-fat diet presented higher bodyweight, blood glucose level, food intake, and water consumption. As 4 weeks of administrated HFD did not induce any obvious insulin resistance, identifying clear symptoms of diabetic rats was required in order to study the inhibitory effect of HC. The combination of a high-fat diets with streptozotocin (STZ) injection in rats was thus set up for inducing late-stage type 2 diabetes [33]. STZ, specifically in beta-cells of the pancreas, generates reactive oxygen species and reactive nitrogen species and causes DNA damage leading to beta-cell dysfunction, decreased insulin biosynthesis and secretion [34, 35]. STZ at a dose of 35 mg/kg.bw successfully-induced diabetic symptoms in HFD-fed rats by increasing blood glucose levels over 250 mg/dL within 72 hours, along with increasing food intake and water consumption. These results indicated that those rats could not use glucose as an energy source of GLUT-4 dependent cells, which subsequently led to the onset of hyperglycemia. Moreover, lower serum insulin levels were observed in HFD-fed/STZ-treated rats. Therefore, the HFD-STZ model, which exhibited impairment of insulin production and glucose utilization, is suitable for determining the antidiabetic efficiency of HC.

By this rat model, administration of HC in diabetic rats, especially at a high dose, could finally improve glucose utilization by reducing blood glucose levels. Additionally, administration of HC could also increase the secretion of insulin from *β*-cells. HOMA-IR is normally used as a parameter to represent insulin sensitivity for glucose uptake into cells. Insulin insensitive shows high levels of insulin and high levels of blood glucose, leading to HOMA-IR increasing. Our results reveal that the combination of HFD and STZ-fed rats elevated HOMA-IR values, which resulted from the low-fasting insulin levels and high-fasting blood glucose levels. However, administration of HC could not significantly reduce the HOMA-IR value, since HC administrated-diabetic rats exhibited the decreasing of fasting blood glucose together with the increasing of insulin levels. By HOMA-IR, it could not be primarily interpreted that HC could decrease blood sugar by increasing insulin levels but not enhancing insulin sensitivity. In agreement with our results, recent meta-analysis of randomized control trials indicated that supplementation with green coffee extract had no significant decreasing effect on HOMA–IR status despite attenuating glycemia [36]. Consistently, it has been reported that while HOMA-IR of diabetic patients that received grape seed extract remained unchanged, grape seed proanthocyanidins can improve pancreatic beta-cells in *in vitro* and *in vivo* studies [37–39]. Thus, the alteration of beta-cell functions and histology was next determined. The impaired beta-cell function in HFD-STZ fed rats was correspondent with the histological examination of the pancreas tissue, which reveals the marked decrease of the Islet of Langerhans area and the number of Islets of Langerhans per pancreas section. Moreover, the expression of proinflammatory cytokine and enzyme genes, including *TNF-α*, *IL-*1*β*, *IL-*6, *iNOS*, *COX-*2, significantly increased in the pancreas of HFD-STZ fed rats, along with noticeable increased oxidative stress and inflammation markers in rats' serum. Proinflammatory mediators have been known to play a role in the progression of type 2 diabetes via beta-cell dysfunction [40, 41]. Exposure of beta-cell to main cytokines, such as IL-1*β*, TNF*α*, and IL-6, induces the generation of reactive oxygen species and the activation of signaling processes that impair beta-cell function. Through our results, it might be suggested that the improvement of pancreas function upon HC administration is partly due to the anti-inflammatory and antioxidant properties of HC. Several anti-inflammatory compounds were thus proposed as therapeutic agents for treating diabetes, since they have the potential ability to preserve beta-cell function [42–44].

In type 2 diabetes, chronic low-grade inflammation in adipose tissue leads to insulin resistance, impaired glucose tolerance, and elevation of free fatty acid in blood circulation [8]. Free fatty acid then contributes to the loss of insulin sensitivity [45]. Palmitate, lipolysis product from insensitive in adipose tissue, can induce insulin resistance in muscles, the liver, and adipose cells by activating the TLR pathway, protein kinase, c-Jun, and NF-*κ*B [46]. It has been reported that 750 *μ*m of palmitate significantly reduced insulin-stimulated glucose uptake through activation of NF-*κ*B and phosphorylation of IRS at Ser 307 and Akt at serine 473 in 3T3L1 adipocytes [47, 48]. In insulin insensitive adipose tissue, lipolysis increases for energy production due to the lack of triglyceride lipase inhibition, leading to the release of free fatty acid and glycerol. The results reveal that the same concentration of palmitate could induce lipolysis in 3T3-L1 adipocytes without affecting cell availability. However, increased intracellular glucose uptake and decreased lipolysis in palmitate-treated 3T3-L1 adipocytes were shown to recover after HCE treatment, which is comparable to diabetic drugs metformin. Phenolic and flavonoid compounds in the extracts may be responsible for hypoglycemic events in both diabetic rats and insulin resistance 3T3-L1 adipocytes. It has been reported that gallic acid attenuates insulin resistance via partial agonism of PPAR*γ* and activates GLUT4 translocation by PI3K/p-Akt signaling pathway [49]. Rosmarinic acid ameliorates hyperglycemia and insulin sensitivity by modulating the expression of phosphoenolpyruvate carboxykinase and stimulating GLUT 4 expression in diabetic rats [50]. In addition, administration of chlorogenic acid in subjects with impaired glucose tolerance could decrease fasting plasma glucose, elevate insulin secretion, and enhance insulin sensitivity [51]. The combination of quercetin with resveratrol suppresses obesity-associated inflammation in rats induced by a high-fat diet via AMPK*α*1/SIRT1 signaling pathway [52]. Apart from bioactive phytochemicals in HC, the product from fermentation mixed in tablets also exhibited the ability to improve insulin resistance and beta-cell functions in *in vitro* studies. Previous studies have demonstrated that lactic acid bacteria-fermented products of green tea and *H. cordata* leaves exert antiadipogenic and antiobesity properties [53]. These bioactive compounds and fermentation products are likely to provide synergistic effects. Nevertheless, the active compounds of *H. cordata* and how their underlying mechanism on insulin resistance is associated with inflammation both need to be further determined.

Previously, Kwon and Ha introduced the possibility of microbial fermentation in elevating major components in plant extract [20]. Fermentation of *H. cordata* carried out on *Bacillus* strains could increase flavonoid contents, such as rutin, quercitrin, and quercetin, which was attributed to the changes in the organic content [20]. Likewise, the present study also demonstrated the presence of high levels of gallic acid and rosmarinic acid in fermented HC. Many studies showed that quercetin [54, 55], rosmarinic acid [56], chlorogenic acid [57], rutin [58], and gallic acid [59] exhibited potential antioxidant, anti-inflammatory, and antidiabetic properties. The other important product from the fermentation by natural microorganism is beta-glucan. It comprises a group of *β*-D-glucose polysaccharides that are naturally occurring in the cell walls of bacteria after fermentation [21]. Experimental evidence has suggested that the consumption of beta-glucan from *Saccharomyces cerevisiae* could decrease blood glucose levels, lipid profile, and plasma atherogenic index in type 2 diabetic rats [60]. In addition, beta-glucan from oats ameliorated insulin resistance by enhancing hepatic glucose kinase activity [61]. Hence, the aforementioned studies on bioactive compounds found in HC fostered our interest to further investigate the modulatory effects of these products on insulin-resistant adipocytes, to relate the concentration of HC in vitro and HCE in vitro.

## 5. Conclusion

Fermented *H. cordata* was found to alleviate diabetic symptoms in rats by recovering beta-cell morphology and function, together with sensitized insulin activation. Additionally, HC was shown to increase the sensitivity of insulin in palmitate-induced insulin resistance 3T3-L1 adipocytes, as reflected by stimulating glucose uptake and reducing lipolysis. Through this study, the scientific data of the fermented *H. cordata* tablets on hyperglycemic rats was reported to support the antidiabetic ability of this commercial product. However, the molecular mechanisms of HC need to be further clarified.

## Figures and Tables

**Table 1:** Relative liver and pancreas weight in each group.

Groups	Bodyweight (g)	Organ weight (g)		Relative organ weight (mg)	
	Final	Liver	Pancreas	Liver	Pancreas
Normal control	457 ± 11	10.3 ± 1.7	2.0 ± 0.2	22.6 ± 3.7	4.4 ± 0.5
HFD control	530 ± 34^++^	14.0 ± 1.3^++^	1.7 ± 0.2	26.7 ± 3.5	3.2 ± 0.3^+^
HFD-STZ control	403 ± 27^+^	13.9 ± 1.4^++^	1.4 ± 0.1^++^	34.8 ± 5.0^++^	3.5 ± 0.6
HC (10 mg/kg)	450 ± 16	13.6 ± 0.6	1.6 ± 0.3	30.0 ± 1.3	3.6 ± 0.6
HC (100 mg/kg)	414 ± 38	12.7 ± 0.3	1.9 ± 0.2^*∗∗*^	30.9 ± 2.8	4.6 ± 0.3^*∗*^
Metformin (100 mg/kg)	373 ± 22	13.2 ± 0.6	1.4 ± 0.2	35.6 ± 3.1	3.7 ± 0.3

Results are presented as means ± SD (*n* = 6 each group). ^+^*p* < 0.05 and ^++^*p* < 0.01 versus normal control group. ^*∗*^*p* < 0.05 and ^*∗∗*^*p* < 0.01 versus HFD-STZ control group.

**Table 2:** The average food intake and water consumption in HFD-STZ-treated rats.

Groups/weeks	Food intake (g/day/rats)^*∗*^		Water consumption (ml/day/rats)^*∗*^	
	0	4	0	4
Normal control	15.4 ± 2.2	17.5 ± 2.2	31.3 ± 6.7	27.5 ± 4.1
HFD control	24.2 ± 3.1^+^	23.8 ± 3.8	32.5 ± 7.5	25.8 ± 3.1
HFD-STZ control	21.7 ± 6.2	35.0 ± 2.0^+++^	57.5 ± 16	77.5 ± 6.1^+++^
HC (10 mg/kg)	22.5 ± 3.8	26.7 ± 5.5^*∗∗*^	53.1 ± 8.5	48.3 ± 16^*∗∗∗*^
HC (100 mg/kg)	24.2 ± 4.5	23.3 ± 3.1^*∗∗*^	55.6 ± 13	53.3 ± 13^*∗∗*^
Metformin (100 mg/kg)	17.5 ± 5.6	26.7 ± 3.4^*∗*^	46.9 ± 5.4	51.7 ± 3.0^*∗∗*^

Results are presented as means ± SD (*n* = 6 each group). ^+^*p* < 0.05, ^++^*p* < 0.01, and ^+++^*p* < 0.001 versus normal control group.^*∗*^*p* < 0.05, ^*∗∗*^*p* < 0.01, and ^*∗∗∗*^*p* < 0.001 versus HFD-STZ control group.

**Table 3:** Levels of FBG, FINS, HOMA-IR, and HbA1c.

Metabolic parameter	FBG (mg/dl)	FINS (ng/ml)	HOMA-IR	HbA1c (%)
Normal control	97.3 ± 9.0	3.2 ± 1.2	0.6 ± 0.4	7.8 ± 0.7
HFD control	116.8 ± 5.0	3.9 ± 2.5	1.1 ± 0.8	9.8 ± 0.7
HFD-STZ control	363.0 ± 21.9^++^	2.8 ± 0.6	2.5 ± 0.5^+^	16.7 ± 1.2^++^
HC (10 mg/kg)	300.7 ± 6.5^*∗*^	5.0 ± 2.8	3.7 ± 2.0	16.3 ± 3.3
HC (100 mg/kg)	244.3 ± 54.7^*∗∗*^	3.34 ± 2.7	2.0 ± 0.9	14.7 ± 2.3
Metformin (100 mg/kg)	305.2 ± 32.3^*∗*^	2.8 ± 2.1	2.1 ± 1.2	16.0 ± 1.3

Results are presented as means ± SD (*n* = 6 each group). ^+^*p* < 0.05 and ^++^*p* < 0.001 versus normal control group. ^*∗*^*p* < 0.05 and ^*∗∗*^*p* < 0.01 versus HFD-STZ control group. FBG: fasting blood glucose; FINS: fasting serum insulin levels; HOMA-IR: homeostasis model assessment of insulin resistance.

**Table 4:** Total cholesterol (TC) and total triglyceride (TG) in each group.

Metabolic parameter	TC (mg/dl)	TG (mg/dl)
Normal control	72 ± 3.4	64 ± 13.7
HFD control	82 ± 12.1	66 ± 10.3
HFD-STZ control	81 ± 10.4^+^	184 ± 62.5^++^
HC (10 mg/kg)	85 ± 6.3	172 ± 28.0
HC (100 mg/kg)	79 ± 12.4	113 ± 22.1^*∗*^
Metformin (100 mg/kg)	81 ± 5.0	167 ± 42.1

Results are presented as means ± SD (*n* = 6 each group). ^+^*p* < 0.05 and ^++^*p* < 0.001 versus normal control group. ^*∗*^*p* < 0.05 and ^*∗∗*^*p* < 0.001 versus HFD-STZ control group.

## Data Availability

The data are available upon request to the corresponding author via e-mail.

## Abbreviations

AUC: Area under the curve
BSA: Bovine serum albumin
CMC: Carboxymethylcellulose
DMEM: Dulbecco's modified eagle medium
ELISA: Enzyme-linked immunosorbent assay
FBG: Fasting blood glucose
FBS: Fetal bovine serum
FINS: Fasting serum insulin levels
GLUT: Glucose transporter
HC: Fermented *Houttuynia cordata* Thunb
HCE: Ethanolic extract of fermented *Houttuynia cordata* Thunb
HCW: Water extract of fermented *Houttuynia cordata* Thunb
HFD: High-fat diet
HOMA-IR: Homeostasis model assessment of insulin resistance
H&E: Hematoxylin and eosin
IBMX: Isobutylmethylxanthine
MDA: Malondialdehyde
NLAC-MU: National laboratory animal center, Mahidol University
PBS: Phosphate buffer saline
SD: Standard deviation
STZ: Streptozotocin
TC: Total cholesterol
TFA: Trifluoroacetic acid
TG: Total triglycerides
2-NBDG: 2-[N-(7-Nitrobenz-2-oxa-1,3-diazol-4-yl)amino]-2-deoxy-d-glucose.
